# Identification of a novel nuclease activity in human DDX49 helicase

**DOI:** 10.1098/rsos.241891

**Published:** 2024-12-18

**Authors:** Ashley J. Parkes, Sabesan Anandavijayan, Anna Lou-Hing, Olivia Downs, Tom Killelea, Louise Martin, Fiorela Kapllanaj, Edward L. Bolt

**Affiliations:** ^1^ School of Life Sciences, University of Nottingham, Nottingham, UK

**Keywords:** novel, nuclease, activity, human, DDX49, helicase

## Abstract

Human DDX49 is an emerging target in cancer progression and retroviral diseases through its essential roles in nucleolar RNA processing. Here, we identify nuclease activity of human DDX49, which requires active site aspartate residues within a conserved region of metazoan DDX49s that is absent from yeast and archaeal DDX49 homologues. We provide evidence that DDX49 nuclease activity is facilitated by its helicase activity. Using CRISPR-Cas9 genetic editing, we show that a heterozygous (*DDX49*^+/−^) U2OS cell line is defective at cell migration, a phenotype supporting the association of DDX49 with cancer cell invasiveness. Measurement of RNAs in *DDX49*^+/−^ indicates that DDX49 is required to sustain levels of 5.8S rRNA.

## 1. Introduction

The DExD box (DDX) protein family is based on conserved amino acid motifs proximal to their ATPase active site (the ‘DExD box’), first described through homologies with RNA helicase eIF4a [1–3]. DDX proteins have since been demonstrated to contribute to RNA processing during transcription, translation, innate immune defence and genome stability in prokaryotes and eukaryotes [4–6]. Human genomes are known to encode 37 DDX proteins—loss or malfunction of individual DDX proteins is associated with diverse human health problems and syndromes [7], although in most cases the precise functions and mechanisms of DDX proteins are not known.

Human DDX49 is emerging as a novel marker and therapeutic target in cancers [7–12], and as a target for anti-retroviral treatment, because it supports replication of human immunodeficiency viruses within human cells [13–15]. DDX49 and its homologue in *Saccharomyces cerevisiae*—Dbp8p—control levels of cytosolic mRNA transcripts and ribosome biogenesis through core DDX amino acid motifs [16–18]. A study of partially purified human DDX49 showed ATP-dependent dissociation of a 10-nucleotide RNA strand from complementary RNA *in vitro* [16].

**Figure 1.**
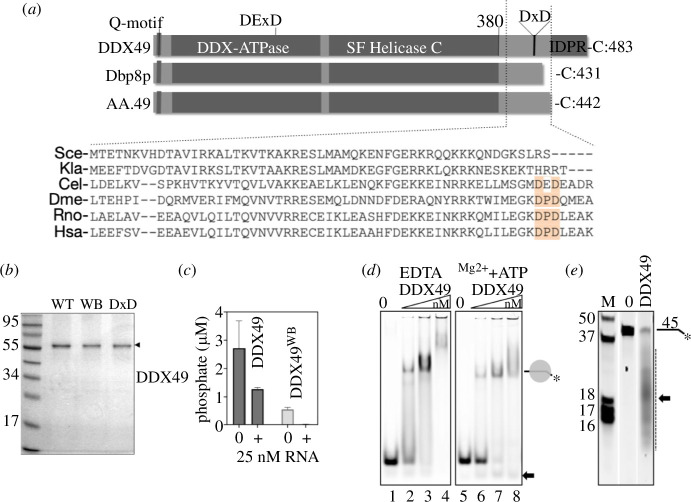
(*a*) Core protein domain similarities between metazoan DDX49 and its homologues from *S. cerevisiae* (Dbp8p), and from an Asgard archaeon (AA.49) that is thought antecedent to eukaryotes [19]. Amino acid sequence alignments of the C-terminal regions revealed a conserved DxD motif and an intrinsically disordered protein region in the metazoan DDX49s. The sequences shown are from *Saccharomyces cerevisiae* (Sce); *Klebsiella lactis* (Kla), bacterial homologue RhlE; *Caenorhabditis elegans* (Cel); *Drosophila melanogaster* (Dme); *Rattus norvegicus* (Rno); and *Homo sapiens* (Hsa). (*b*) Purified DDX49 proteins (5 μg each) Coomassie-blue stained with size marker (kDa). (*c*) Malachite green reporter measurements of DDX49 and DDX49^WB^ (500 nM) ATPase activities in the presence or absence of an RNA oligonucleotide at 25 nM. Plots are from three independent measurements, with bars of the standard error of the mean. (*d*) EMSAs of DDX49 (0, 200, 500 nM and 1 μM) binding to an RNA oligonucleotide (25 nM) as a stable complex in reaction buffers and gels containing either EDTA (lanes 1–4) or 0.1 mM each of magnesium and ATP (lanes 5–8). The arrow points to potential nuclease activity in low ATP-Mg^2+^ concentration. (*e*) RNA nuclease products from DDX49 (800 nM) mixed with an RNA oligonucleotide (25 nM) compared with a no protein control (0), in a urea TBE gel alongside a nucleotide size marker ladder. Shown are individual lane slices from a much larger single gel of multiple reactions, which is shown in full in electronic supplementary material, figure S1.

A feature of metazoan DDX49 proteins that is absent from yeast Dbp8p, is a C-terminal region of about 50 amino acids, summarized in figure 1. To investigate the characteristics of this C-terminal region, we purified the human DDX49 protein and multiple mutant forms. We report that nucleic acid binding and duplex unwinding by human DDX49 are coupled with a novel nuclease activity that requires a Toprim-like motif [20,21] in the C-terminal region. We were unable to generate fully deleted *DDX49* human U2OS cells using CRISPR-Cas9, consistent with the essentiality of DDX49 in Human Cancer Dependency Mapping [22], and demonstrated for Dbp8p in *S. cerevisiae* [17]. However, *DDX49*^−^/*DDX49*^+^ heterozygotes showed reduced cellular migration and 5.8S rRNA processing.

## 2. Results and discussion

### 2.1. Identification of a novel nuclease active site in human DDX49 that requires the known DDX49 helicase activity

Core conserved domains of DDX49 proteins are typical of RecA-like ATPases and superfamily-2 helicases, but metazoan DDX49 proteins have an extended C-terminal region that contains a conserved Asp-X-Asp motif (where X is a variable amino acid) followed by an intrinsically disordered protein region (IDPR) (figure 1*a*). To investigate this region, we purified the wild-type human DDX49 protein and mutant forms for biochemical assays (figure 1*b*)—initial mutations were in the core DDX ATPase ‘Walker B’ motif (DDX49^D152A+D155A^, herein called DDX49^WB^) and the Asp-X-Asp motif (DDX49^D422A-D424A^, herein called DDX49^DxD^).

**Figure 2.**
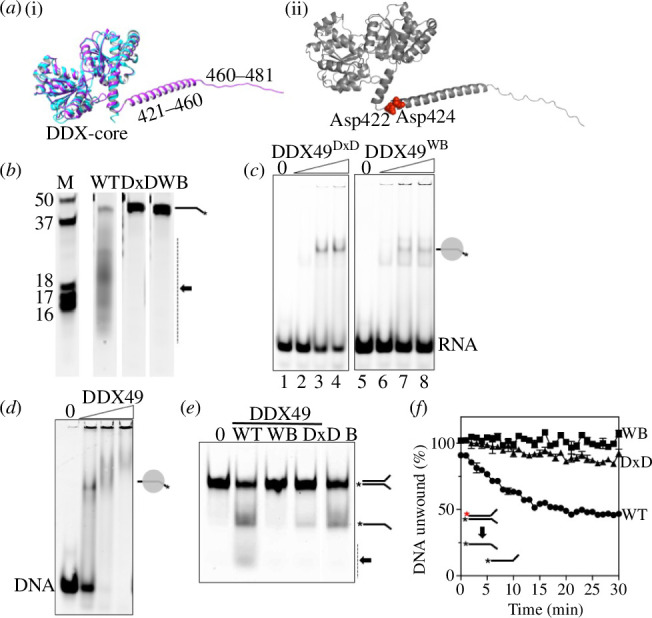
*(a*) (*i*) AlphaFold prediction of human DDX49 protein structure (cyan) superimposed on the *S. cerevisiae* homologue Dbp8p (blue). It exposes the C-terminal alpha-helix and IDPR amino acid regions of human DDX49. (ii) Highlighting (red, space-fill) the human DDX49 predicted Asp-422 and Asp-424 (DxD) motif. (*b*) Urea gel slices showing RNA nuclease activity from 500 nM of wild-type DDX49 (arrowed) compared with no detectable activity from the DDX49^WB^ (WB) and DDX49^DxD^ (DxD) mutant proteins—the panel shows individual lane slices from a single image of the single large gel in electronic supplementary material, figure S1. (*c*) EMSAs of binding DDX49^DxD^ and DDX49^WB^ mutants (0, 200, 500 nM and 1 μM) to an RNA oligonucleotide (25 nM) in 0.1 mM of magnesium and ATP. (*d*) EMSA of binding DDX49 (0, 200, 500 nM and 1 μM) to a DNA oligonucleotide (25 nM) equivalent to the RNA oligonucleotide in 0.1 mM of magnesium and ATP. (*e*) Helicase and nuclease products from DDX49 proteins as indicated (wild type, WT; Walker B mutated, WB; nuclease Asp-x-Asp mutated, DxD) in reactions containing 5 mM each of magnesium and ATP and a forked DNA substrate that is unwound to cy5-labelled ssDNA and nuclease processed to the product indicated by the arrow and dotted line. Lane marked B is for a boiled reaction, from the same gel but with intervening irrelevant lanes removed for this image. (*f*) FRET measurements of DNA helicase unwinding from 500 nM of DDX49 WT compared with the WB and DxD mutant proteins as indicated on the graph panel, showing mean values of two repeats and error bars of standard deviation.

ATPase activity of DDX49 (500 nM) was inhibited by the addition of the 45-nucleotide RNA oligonucleotide used at either 25 nM or 2.5 μM (figure 1*c* and electronic supplementary material, S1), the same effect of RNA on DDX49 observed in a previous study [16]. DDX49 forms a stable nucleoprotein complex with RNA in EMSAs containing either EDTA or 0.1 mM of magnesium and ATP added to the EMSA gel and buffers (figure 1*d*). The magnesium-ATP EMSAs at the highest concentration of DDX49 (1 μM) (figure 1*d*, lane 8) showed weak potential nuclease activity, which became clear when repeated using 5 mM magnesium and ATP, and then reactions deproteinized and electrophoresed through urea gels (figure 1*e* and electronic supplementary material, figure S2). To validate that the RNase activity was catalysed by DDX49 rather than a contaminant in purification from *Escherichia coli*, we selected the DDX49 C-terminal Asp-X-Asp motif (human DDX49 Asp-422 and Asp-424) for mutation to Ala-X-Ala (DDX49^DxD^) (figure 1*a*). This motif is present only in the metazoan DDX49s, where it is preceded by a pattern of hydrophobic residues that together resemble a nuclease Toprim motif [20,21,23]. Further evidence supporting this arose from AlphaFold [24] modelling of human DDX49, which predicted (with local confidence <70 pLDDT ≤90/100) the C-terminal region of human DDX49 comprising an alpha-helix (figure 2*a*(i)) containing proximal orientation of Asp-422 and Asp-424 side chains, consistent with an active site (figure 2*a*(ii)).

DDX49^DxD^ and DDX49^WB^ proteins (figure 1*b*) gave no RNA nuclease product compared to wild-type DDX49 (figure 2*b*). DDX49^DxD^ and DDX49^WB^ both formed stable nucleoprotein complex with RNA in EMSAs, when used at the same concentration as in the nuclease assays (500 nM, figure 2*c*), confirming that loss of nuclease activity is not caused by inability to bind RNA. To compare directly the nuclease and helicase activities of DDX49, DDX49^DxD^ and DDX49^WB^, we visualized products in endpoint assays and using FRET in real time. A DNA fork substrate was used, enabling us to visualize helicase unwinding and nuclease activity in the same reactions, because although DDX49 binds to DNA stably (figure 2*d*) and can also unwind fork DNA (figure 2*e,f*) it has much reduced DNase activity compared with RNase activity on oligonucleotides of equivalent sequence (electronic supplementary material, figure S3). Helicase and nuclease products observed for wild-type DDX49 were undetectable using DDX49^WB^, and DDX49^DxD^ gave much-reduced helicase product without nuclease product (figure 2*e*). FRET readouts were consistent with the gel products, showing inactive DDX49^WB^ alongside very weak activity from DDX49^DxD^ (figure 2*f*).

We conclude that DDX49 nuclease activity requires the C-terminal Asp-X-Asp motif. Although this can process RNA and DNA, in common with Toprim-family nucleases more generally [21], it is most reactive to RNA, explainable by the precise disposition of the active site aspartates, water and magnesium towards RNA sugar–phosphate chains, compared with DNA. The ability of DDX49 to unwind RNA [16] and DNA is shared with other DDX-family helicases, at least when studied *in vitro* [25–27], though it differs from RNA-specific translocation exemplified by Vasa-family helicases [28]. Within cells, recruitment of DDX proteins to specific RNA processing complexes would provide RNA targeting, recently reviewed in [29] and may stimulate DDX49 ATPase activity to degrade RNA, since we and others [16] have shown that ATPase activity of isolated DDX49 protein is inhibited by RNA. Much decreased helicase activity from DDX49^DxD^ (figure 2*e*,*f*), despite possessing intact ATPase/helicase domains, tallies with lack of nuclease activity from ATPase defective DDX49^WB^ (figure 2*b*), which has intact the DxD motif. This indicates functional interdependence of the C-terminal nuclease region with core DDX ATPase domains. Further support emerged from removing the DDX49 C-terminal region, described next.

### 2.2. Human DDX49 C-terminal intrinsically disordered protein region is required for RNA binding

**Figure 3.**
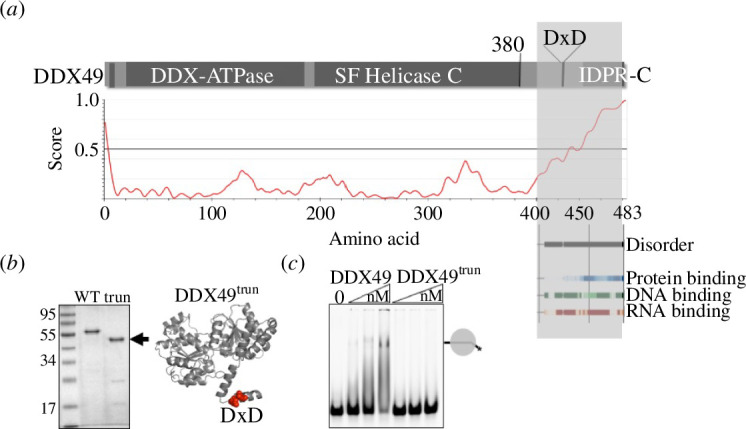
(*a*) Schematic mapping of human DDX49 protein to database predictions for the metazoan-specific DDX49 C-terminal region (shaded grey) and including predicted regions of DNA and RNA binding and also predicted protein binding. Also shown is the plot predicting IDPR, in the C-terminal region with a value of >0.5. (*b*) Purified DDX49 compared with DDX49^trun^ (5 mg each) after Coomassie-blue staining, with a cartoon to highlight the region of DDX49 that was deleted compared with figure 2*a*. (*c*) EMSA of binding DDX49 and DDX49^trun^ (0, 200, 500 nM and 1 μM) to RNA (25 nM) in EDTA.

AlphaFold, flDPnn [30] and IUPred3 [31] predicted that an IDPR at the human DDX49 C terminus may bind nucleic acids, summarized in figure 3*a*. To test this, we purified DDX49 protein without the extended C-terminal region (called DDX49^trun^), lacking the C-terminal 51 amino acids (residues 432–483) that include the predicted IDPR, but retaining the nuclease active site residues (figure 3*b*). DDX49^trun^ was unable to form stable RNA complex in EMSAs (figure 3*c*), supporting bioinformatics predictions, although this raises the question of how yeast and archaeal DDX49 proteins bind to RNA, given that they lack this IDPR region. We speculate, in the absence of experimentally determined structures for DDX49, that metazoan DDX49 proteins modulate RNA binding through physical interaction between residues of the DDX49 C-terminal helical region and the DDX49 core domains, and that this also controls coupled nuclease and helicase activity observed in figure 2.

### 2.3. Human DDX49 promotes cell migration and processing of 5.8S rRNA

**Figure 4.**
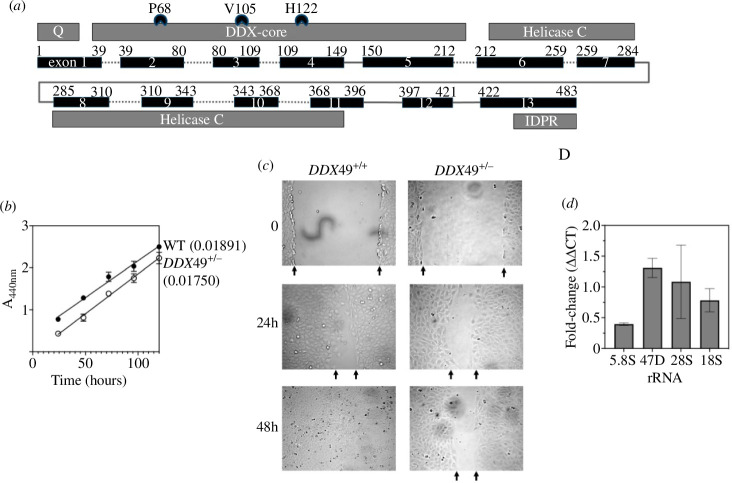
(*a*) Exon organization of human *DDX49* with positions of CRISPR-Cas9 sites tested (codons P68, V105 and H122). Targeting of site V105 successfully generated the heterozygous U2OS cell line (*DDX49*^+/−^), detailed in §3. (*b*) WST-1 cell proliferation measurements (A_440nm_) comparing *DDX49*^+/−^ with *DDX49*^+/+^ U2OS cells. Each measurement is labelled with the equation for the slope of the line. The graph shows means with standard deviation error bars from three repeats. (*c*) Images of a representative wound-scratch healing assay done in triplicate comparing the rate of migration of *DDX49*^+/−^ and *DDX49*^+/+^ U2OS cells from 0 to 48 hours, with arrows indicating the edge of migrating confluent cells. Images were captured using phase-contrast microscopy at ×10 magnification. (*d*) RT-qPCR measurements of pre-rRNA levels in *DDX49*^+/−^ cells compared with levels in *DDX49*^+/+^ cells that were assigned a value of 1.0; the relative target rRNA expression was calculated using the comparative Ct (DD CT) method and the fold change of rRNA expression calculated using the formula 2^−ΔΔ^*^CT^*. The values were calculated as a mean of three independent experiments and show error bars for standard deviation.

Genome-wide CRISPR-Cas9 and RNAi depletion studies suggest that human DDX49 is a common essential gene—DDX49 depletion data can be found at the DepMap portal (https://depmap.org/portal/). Consistent with this, when we targeted *DDX49* exons 2, 3 and 4 with gRNAs for CRISPR-Cas9 knockout in human osteosarcoma (U2OS) cells (figure 4*a*), only heterozygous cells (*DDX49*^+/−^) could be recovered—see §3 for a full description of their detection and validation. Attempts to use ‘Prime’ editing (nCas9-RT [32]) of U2OS cells to generate DDX49 point mutations targeted to the Asp-422 and Asp-424 codons were also consistent with lethality, as no viable cells were generated compared with the control Prime editing reactions that were viable.

We established a heterozygous *DDX49*^+/−^ U2OS cell line with a 19-nucleotide deletion within one copy of DDX49 exon 3. This was used to measure cell proliferation and migration compared with *DDX49*^+/+^ U2OS cells. Cell proliferation measured by WST-1 showed no change in growth rate when comparing absorbance readings of *DDX49*^+/−^ and *DDX49*^+/+^ cells, measured over 100 hours of cell culture (figure 4*b*). However, in a scratch-wound healing assay [33], the *DDX49*^+/−^ cell migration was slowed compared with *DDX49*^+/+^ cells—in each of three replicate experiments, the migration of *DDX49*^+/+^ cells to heal the scratch was complete by 48 hours but was not for *DDX49*^+/−^ cells, summarized in figure 4*c*. This supports that human *DDX49* is required for cell migration, and studies in which knock-down of *DDX49* by RNAi (using lentiviral encoded shRNAmiRs) resulted in reduced invasiveness of prostate and lung cancer cells [8,12], and reduced colony-forming efficacy in HeLa cells [16]. The prior work using RNAi depletion of DDX49 in HeLa cells [16] indicated that DDX49 is required to process pre-ribosomal rRNA in the nucleolus. We therefore compared rRNA levels in the *DDX49*^+/−^ U2OS cells with *DDX49*^+/+^ cells using RT-qPCR, against *GAPDH* as a control. This revealed that 5.8S rRNA levels were significantly reduced in *DDX49*^+/−^ cells compared with *DDX49*^+/+^ cells, with no significant change in 47S, 28S and 18S rRNAs (figure 4*d* and electronic supplementary material, figure S4). Defects in cellular 5.8S rRNA processing cause defects in protein elongation during translation and reduced cellular proliferation [34–36], the latter consistent with a role for 5.8S rRNA processing by DDX49, manifest in cell proliferation defects of DDX49^+/−^ cells. Interestingly, over-expressing DDX49 has also been reported to increase the proliferation of HeLa cells, an effect that was suggested could result from unidentified functions of DDX49 [16].

Given the link between DDX49 and progression of human diseases, identification of a novel nuclease activity and the importance of the C-terminal IDPR may provide new means to investigate the targeting of DDX49 in cells.

## 3. Methods

### 3.1. Generation and purification of DDX49 proteins

The 1449 nucleotide ORF encoding human DDX49 (CCDS: 12390.1) was optimized for expression in *E. coli*, synthesized and inserted into pET100/D-TOPO by GeneArt (Life Technologies). Plasmids used in the work are listed in electronic supplementary material, table S1; in brief, each was constructed via site-directed mutagenesis of DDX49 expression vector using primers listed in electronic supplementary material, table S2. Successful mutants were confirmed through Sanger sequencing.

Expression of DDX49 and mutants was carried out in *E. coli* strain BL21 AI (Invitrogen) transformed with the appropriate plasmid. Individual colonies were used to prepare fresh cultures overnight with shaking at 37°C in Luria–Bertani (LB) broth containing ampicillin (0.1 mg ml^−1^), prior to dilution (1 : 100) into fresh LB and ampicillin. Cultures were grown at 37°C to OD_600_ 0.6 prior to induction of protein expression by the addition of 0.5 mM IPTG (isopropyl β-ᴅ-1-thiogalactopyranoside) and 0.1% (v/v) ʟ-arabinose. Overexpression was carried out for 16 hours at 18°C with shaking.

Purification was at 4°C unless stated. Biomass was recovered by centrifugation (5000*g*, 10 min), resuspended in buffer A (25 mM Tris–HCl pH 7.5, 500 mM NaCl, 25 mM imidazole, 10% glycerol) and supplemented with 1 mM PMSF. Cells were lysed by sonication, lysate cleared (35 000*g*, 35 min) and soluble fraction was loaded onto a 5 ml HisTrap^TM^ HP column (GE Healthcare) at ambient temperature prior to elution in a gradient of 25–400 mM imidazole. DDX49 fractions were confirmed through SDS-PAGE, pooled and dialysed into pre-chilled buffer B (25 mM Tris–HCl pH 7.5, 200 mM NaCl, 10% glycerol) for 16 hours. Dialysate was loaded onto a HiTrap Heparin HP column (1 ml) for further purification over a 0.2–1.0 M NaCl gradient. DDX49 fractions were identified, pooled and dialysed as previously described into buffer C (25 mM Tris–HCl pH 7.5, 200 mM NaCl, 35% glycerol and 2 mM DTT). DDX49 proteins were aliquoted and snap-frozen using dry ice prior to storage at −80°C. Protein concentrations were determined from UV absorption readings (280 nm) with a DeNovix nanodrop spectrophotometer using the Beer–Lambert law and an extinction coefficient of 27 350 M^−1^ cm^−1^.

### 3.2. Assays and substrates

RNA and DNA strands for *in vitro* assays using purified proteins were custom synthesized (Sigma Aldrich) and are listed in electronic supplementary material, table S3. ATPase activity was measured using malachite green colorimetric detection of phosphate generation in a BIOMOL Green kit (Enzo Life Sciences). FRET measurements for helicase assays used Cy5 and Cy3 end-labelled DNA to form a FRET pair to observe decreasing energy transfer during substrate unwinding. This was measured as changes in sample emission at 590 nm in a pre-warmed (37°C) FLUOstar Omega microplate reader (BMG Labtech) at 1 min intervals for 30 min. Gains were adjusted to the highest signal (Cy3 control) prior to the addition of protein. Data were plotted using Prism software.

Gel-based assays used Cy5 end-labelled RNA or DNA imaged using an Amersham Typhoon 5 biomolecular imager (laser LD635, filter-set Cy5 Fltr 670BP30). EMSAs incubated DDX49 proteins with RNA or DNA (25 nM) in buffer HB (20 mM Tris–HCl pH 7.5, 7% glycerol, 100 µg ml^−1^ bovine serum albumin) supplemented with 25 mM DTT and EDTA (1 mM) or magnesium chloride and ATP (0.1 mM each) as stated, for 30 min at 37°C prior to direct loading on a gel comprising 5% acrylamide in TBE buffer, or TB buffer supplemented with magnesium chloride and ATP (0.1 mM each) for electrophoresis in a Protean II tank (Bio-Rad) for 90 min at 6 watts.

Gel-based nuclease activity assays were in buffer HB supplemented with 5 mM ATP, 5 mM magnesium chloride and 25 mM DTT, incubating DDX49 proteins with Cy-5 end-labelled nucleic acid (25 nM) for 30 min at 37°C prior to stopping the reaction by addition of proteinase K as a 2 mg ml^−1^ solution in 2.5% SDS and 200 mM EDTA. Reactions were visualized on 10% native TBE gel following 60 min migration, or in the case of denaturing gels, a 15% acrylamide TBE gel containing 7 M urea and migration at 10 watts for 180 min in formamide dye (15% w/v formamide; 4 mM EDTA; 4% v/v glycerol).

### 3.3. Cell lines and culture conditions

U2OS and derived cell lines used within this study were routinely cultured at 37°C under 5% CO_2_ in Dulbecco’s modified Eagle medium (DMEM) supplemented with 10% fetal bovine serum, 2 mM ʟ-glutamine and 1% PenStrep (100 U ml^−1^ penicillin and 100 μg ml^−1^ streptomycin), hereafter referred to as complete medium. All experiments were carried out at low passage, and all plasmids used within transfections were purified endotoxin-free using ZymoPURE II Plasmid Maxiprep kit (Zymo Research), according to manufacturer instructions.

### 3.4. CRISPR-Cas9 genetic editing of *DDX49*

The Alt-R™ CRISPR-Cas9 workflow from Integrated DNA Technologies (IDT) was used to target exons in *DDX49* of human U2OS-derived cells. Suitable crRNA sequences for generating gRNAs targeting *DDX49* exons were predicted using the IDT RNA design checker tool, each with a 5′-TGG PAM:

Exon 2: 5′AAGCTGTCTGAGGATCCCTA

Exon 3: 5′AAAGACTGCATCATCGTCGG

Exon 4: 5′GCTCTCTCGGAAACCACACG

Cells were cultured in complete medium to 80% confluency, treated with trypsin, and following a cell count in triplicate were seeded 24 hours prior to transfection at a density of 40 000 cells per well in 24-well plates. The gRNAs were formed by annealing the crRNA with tracrRNA following the manufacturer’s protocol and mixed with recombinant Alt-R HiFi Cas9 nuclease in Cas9Plus reagent and serum-free media to form a ribonucleoprotein targeting complex (RNP, 240 nM). Each RNP was tested individually and transfected into cells using lipofectamine™ CRISPRMAX™ (ThermoFisher) containing gRNA-Cas9 mix to a final concentration of 11 nM RNP per well. A plasmid expressing enhanced green fluorescent protein was used as a transfection control.

Cells were incubated for 48 hours prior to detachment, counting and seeding into two 96-well plates at a density of 0.5 cells per well. Plates were incubated undisturbed at 37°C under 5% CO_2_ for one week, and single colony-forming units were identified using an Axiovert S 100 microscope and expanded into larger culture vessels. Editing efficiency was checked throughout the workflow; genomic DNA was extracted using a PureLink™ Genomic DNA Mini Kit according to the manufacturer’s protocol and quantitated using a DeNovix spectrophotometer, using dsDNA concentration = 50 μg ml^−1^ × OD260 × dilution factor. Using primer sequences flanking the editing sites, designed by CRISPOR [37], genomic DNA (100 ng) was PCR amplified and amplicon products denatured and annealed to form heteroduplexes. Products were digested with T7 endonuclease 1 for 1 hour at 37°C prior to visualization through 2% agarose gel electrophoresis. Amplicons positive for heteroduplex formation were confirmed by Sanger sequencing; edited amplicons were compared with wild-type amplicons using TIDE analysis suite (http://shinyapps.datacurators.nl/tide/) with parameters set to identify Indels with a maximum size of 50 bp and a *p*-value threshold of 0.001. Successfully edited cells were transferred to vapour-phase liquid nitrogen for long-term storage.

### 3.5. Prime editing of DDX49

Prime editing of the *DDX49* D422-x-D424 motif used the prime editor 2 (PE2) expression vector (Addgene number 169850) transfected alongside modified versions of the sgRNA (Addgene number 65777) and prime editing guide RNA (pegRNA, Addgene number 132777) expression vectors. The sequences used to construct the guide RNA plasmids were designed using pegFinder tool [38] and are given in electronic supplementary material, table S4.

### 3.6. U2OS cell proliferation and migration assays

Cell proliferation was assessed using WST-1 cell proliferation reagent (Sigma Aldrich) at 24, 48, 72, 96 and 120 hour time-points. In summary, 4000 cells per well were seeded into 96 well plates and incubated for 24 hours at 37°C under 5% CO_2_. WST-1 reagent (10 ml) was added at time-points and plates were incubated for four more hours. Absorbance was recorded at 440 nM using a FLUOstar Omega microplate reader. Background absorbance was corrected using wells containing media only and cell growth rate assessed relative to Δabsorbance. For migration assays, cells were cultured in 100 mm culture dishes until 90–100% confluency. A scratch in the monolayer was introduced via a sterile micropipette tip, cells were washed with PBS and imaged (Axiovert S100 microscope, Zeiss). Cells were incubated at 37°C and 5% CO_2_ and imaged.

### 3.7. Quantification of rRNA

Total RNA was extracted from cell lines using RNeasy mini kit (Qiagen) to generate cDNA using superscript IV (Thermo Fisher) and oligo (dT)20 (Thermo Fisher), oligos used according to the method in [39], in a GuardOne laminar flow cabinet (Starlab). cDNA was incubated with RNase H (5 units, New England Biolabs) at 37°C for 20 min to remove residual RNA from the sample. Primers specific to beta-actin, UBE2, GAPDH and the rRNAs 47S, 28S, 18S and 5.8S were designed as in electronic supplementary material, table S5. UBE2 and GAPDH were housekeeper controls. PCR amplifications were in 20 μl reaction solutions consisting of 1× SYBR Green Master Mix, 500 nM of each primer and 5 ng of cDNA. Primer specificity was examined through melting curve analysis (95°C for 1 s, 60°C for 20 s and a rise in temperature to a ramp rate of 0.15°C s^−1^). To confirm reproducibility, each assay was performed with technical triplicates for each of the three biological samples. Relative quantification of qPCR data was carried out using the comparative cycle threshold (CT) method, as defined by the following equations:


ΔCT=CT(rRNAspecies)−CT(GAPDH),

$$\Delta \Delta CT=\Delta CT\;(DDX49^{+/-}cells)-\Delta CT\;(wild\;type\;U2OS\;cells).$$

The fold change of target rRNA expression was then calculated using the formula 2^−ΔΔ^*^CT^*, with relative expression of reference samples set at 1.

## Data Availability

Supplementary and raw data files are available on Dryad [40]. Supplementary material is available online [41].
